# Island-Wide Surveillance of Gastrointestinal Protozoan Infection on Fiji by Expanding Lymphatic Filariasis Transmission Assessment Surveys as an Access Platform

**DOI:** 10.4269/ajtmh.17-0559

**Published:** 2018-02-05

**Authors:** Sung Hye Kim, Milika Rinamalo, Meleresita Rainima-Qaniuci, Nemani Talemaitoga, Mike Kama, Eric Rafai, John H. Lowry, Min-Ho Choi, Sung-Tae Hong, Jaco J. Verweij, Louise Kelly-Hope, J. Russell Stothard

**Affiliations:** 1 Department of Parasitology, Liverpool School of Tropical Medicine, Liverpool, United Kingdom;; 2 Ministry of Health, Dinem House, Suva, Republic of Fiji;; 3 School of Geography, Earth Science, and Environment, The University of South Pacific, Suva, Republic of Fiji;; 4 Department of Parasitology and Tropical Medicine, Seoul National University College of Medicine, Seoul, Republic of Korea;; 5 Laboratory of Medical Microbiology and Immunology, Elisabeth Hospital, Tilburg, The Netherlands

## Abstract

As part of lymphatic filariasis (LF) transmission assessment surveys (TAS) on Fiji, an island-wide assessment of gastrointestinal protozoan infection was performed by inspection of a concomitant stool sample collection to investigate the distribution of parasitic protozoa. All grade 1 and 2 students of 69 schools on the two main islands were targeted in two phases (one in the Western Division and the other in the Central and Northern Divisions, except Taveuni sub-Division of Northern), where fecal samples of 1,800 students were available for coproscopy using formalin-ether-acetate concentration. The overall prevalence of *Giardia* infection was 1.6%, having 2.2% in Western and 0.8% in Central/Northern Divisions (*P* = 0.094). The school-level prevalence of giardiasis ranged from 0% to 15.4%, and hotspot analysis using the Getis-Ord Gi* method detected spatial heterogeneity of giardiasis prevalence in schools around Lautoka (*Z*-score = 3.36, *P* value < 0.05), an area affected by Cyclone Kofi in February 2014. Any protozoan infection prevalence was 4.9% in Western and 4.4% in Central/Northern Divisions (*P* = 0.825). Real-time polymerase chain reaction analysis to confirm the findings from a parasitological examination of a 10% stool archive in 95% ethanol from Western Division revealed an elevated prevalence of giardiasis up to 22.4%, the presence of *Entamoeba histolytica*, and the absence of *Cryptosporidium parvum*. Obtaining stool samples alongside LF TAS is a convenient access platform for cosurveillance of gastrointestinal protozoan infection and has pinpointed hitherto unknown hotspots of giardiasis in urban city centers of Fiji. This calls for greater attention to apply tailored water, sanitation and hygiene measures for the control of these parasites.

## INTRODUCTION

Oceania is a region of tropical and subtropical islands in the Pacific Ocean where one-quarter of the population is living in poverty, which places them at an increased risk of several neglected tropical diseases.^1^ Among others, lymphatic filariasis (LF) and soil-transmitted helminthiasis (STH) are particularly widespread.^2^ However, regarding gastrointestinal protozoan infection, such as giardiasis, their epidemiology is not well known,^1,3^ outside Australia,^4,5^ New Caledonia,^6^ New Zealand,^7,8^ and Papua New Guinea,^9^ even if the infection is associated not only with acute and self-limiting illnesses but also chronic diseases such as persistent diarrhea and malabsorption.^10,11^

Fiji is an island country with the fourth largest population in the Pacific, where up to 835,000 Fijians reside mostly on two main islands out of 100 consistently inhabited.^12^ Throughout the country, there has been a long history of efforts made against LF and STH,^2^ but the occurrence of gastrointestinal protozoan infection is only scantily documented and not received sufficient attention, despite inadequate sanitation and safe water coverage at the national level.^13^ The infection may be persistent and as a major public health problem in this part of the world, especially in rural areas,^14^ where water and sanitary conditions are worse. Previous surveillance efforts have attempted to determine the burden of the gastrointestinal protozoan infection in a few pockets of the country^15,16^ but has likely underestimated its true prevalence, as solely insensitive microscopic methods such as direct fecal smear were used.^17^

To this end, we designed a cross-sectional population-based survey using the LF transmission assessment survey (TAS) as a surveillance platform, with the aim of providing comprehensive information on the extent and distribution of gastrointestinal protozoan infection on Fiji. The study targeted primary school students across two main islands of the country, with different demographic characteristics, and examined their infection status using coproscopy.

## MATERIALS AND METHODS

### Study area.

**Figure 1.**
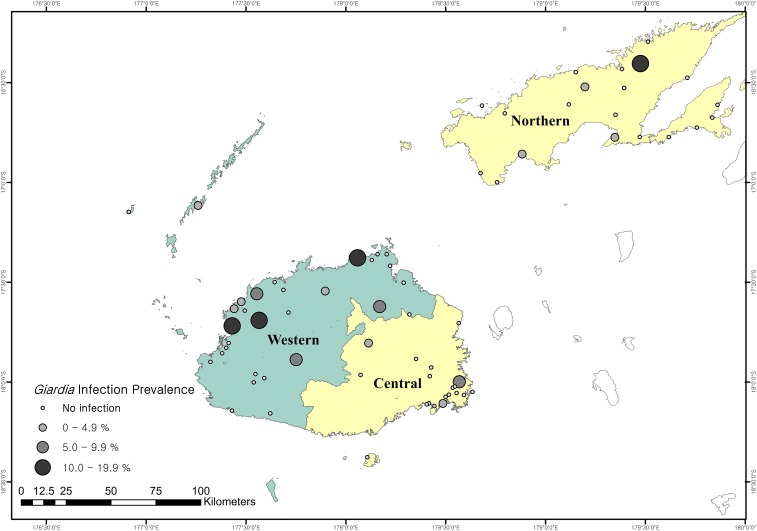
Sketch map of 69 surveyed schools and the school-level prevalence of *Giardia* infection by the formalin-ether-acetate concentration technique in the study area. This figure appears in color at www.ajtmh.org.

The survey was conducted on two main islands of Fiji, Viti Levu and Vanua Levu, following the predetermined schedule of LF TAS in three Divisions that are equal to three LF evaluation units (EUs): the first survey took place in the Western Division in February 2014, which is the dry western half of Viti Levu, and the next in the Central and Northern Divisions except Taveuni sub-Division (Figure 1) from late 2014 to February 2015, which is another wet half of Viti Levu and the whole of Vanua Levu. Ecologically, the Western Division is further divided into a strong dry zone in the western half and a moderate dry zone in the eastern half, whereas the Vanua Levu (Northern Divisions except Taveuni sub-Division) is divided into its dry north and wet south.^18^

### Study design and sampling strategy.

There are 585 primary schools registered at the Ministry of Education in the survey areas, and 77, 82, and 50 schools were selected, respectively, for TAS in three LF EUs, namely, Western, Central, and Northern (except Taveuni sub-Division) Divisions using the Survey Sample Builder (The Task Force for Global Health, Atlanta, GA), based on the World Health Organization’s (WHOs) guidelines for assessing the impact of mass drug administration against LF^19^ in areas with *Aedes* spp. as a principal vector. In Western Division, as the area is further divided into two ecological zones, but schools in the moderate dry zone are all rural, a total of 30 schools were subsampled for this study. In Central/Northern Divisions, we simply subselected 10 rural and 10 urban schools in each LF EU from the list of preselected schools for TAS. Consequently, the estimated sample size of Western and Central/Northern Divisions in this study was 1,692 and 2,203, respectively.

### Data collection procedure and specimen examination.

Two teams visited schools for the period of 2–3 weeks for each TAS and performed stool sample collection in conjunction with TAS procedures. School locations were classified either as urban or rural after the Ministry of Education’s designation (most urban schools are in city councils) of schools, and their main water source and type of latrines were identified using a predefined questionnaire by the surveyors. Mouthed screwed-capped stool containers were distributed in advance to each of first- and second-grade students together with the consent form to be reviewed by their parents. On the survey date, before finger prick for LF antigen testing and measurement of their weight and height, students were asked to submit a fresh morning stool. Then, stool samples in cooler boxes were transported by car, boat, or plane on that day to the national parasitology reference laboratory in Suva.

Regarding a prospective screen for protozoan infection using locally available resources, the formalin-ether-acetate concentration (FEC) technique^20^ was performed for the detection of parasitic protozoa from all available stool samples, and a direct iodine wet preparation was used to enhance the detail of protozoan cysts. Seeking a more precise appraisal, for the stool samples collected in the Western Division survey, an aliquot of approximately 500 mg of stool was filtered through a 212-micron metal sieve, then preserved in 95% ethanol, and was assessed by molecular diagnostics. A systematic subsample (every 10th sample) of the Western Division samples were then transported to the Netherlands for examination by real-time polymerase chain reaction (PCR) with TaqMan^®^ hydrolysis probes, as described previously.^21,22^

### Data management and analysis.

The data collected were entered into an Excel spreadsheet and double checked by project officers of the Fiji Center for Communicable Disease Control. Anthropometric indices adapted 1) height-for-age *z*-score (HAZ) to assess stunting, 2) body mass index for age *z*-score (BAZ) to assess wasting, and 3) weight-for-age *z*-score (WAZ) to assess underweight, using WHO AnthroPlus software version 1.0.4 (WHO, Geneva, Switzerland). The values were expressed as differences from the median in standard deviation (SD) units (i.e., *z*-scores). Participants were classified as stunted, wasted, and underweight if *z*-scores of the HAZ, BAZ, and WAZ were less than two SDs below the National Center for Health Statistics references/WHO median. The STATA Release 14 (StataCorp LP, College Station, TX) was used for statistical analysis. The 95% confidence intervals (CIs) for prevalence were calculated using the CI calculator (available at: http://vl.academicdirect.org/applied_statistics/binomial_distribution/ref/CIcalculator.xls). The χ^2^ test was used to compare the difference in prevalence, and the level of significance was set at 5%.

The coordinates of surveyed schools were collected using a handheld global positioning system device, and the location was confirmed by use of Google Earth. All data were imported into geographic information systems software ArcGIS version 10.2 (ESRI, Redlands, CA) for mapping and spatial analysis. First, the different prevalence distribution of the *Giardia* infection across the surveyed area was mapped. Second, hotspot analysis of the prevalence of giardiasis was conducted using ArcGIS 10.2 Spatial Statistics tools (ESRI). The Getis-Ord Gi* statistic was used to identify the specific locations where high and low prevalence levels were clustered (*Z*-scores, 95% CI +1.96 and −1.96 SDs). In addition, the kernel density estimation method, a nonparametric way of estimating a probability surface using a Gaussian probability density function, was used to create a continuous surface representing the high-to-low prevalence distributions of *Giardia* infection.

### Ethical consideration.

The study was approved by the Fiji Ministry of Health and Medical Services National Health Research Committee and the Ethical Review Board of Liverpool School of Tropical Medicine (14-01). Participation was fully voluntary, and parents were requested to sign a consent form, which was provided in three local languages if they would like their children to take part in the study. Children were allowed to opt out at any time during the survey.

## RESULTS

In total, 932 students in 30 schools from Western and 958 in 39 schools from Central/Northern Divisions, except one special school for the disabled in Central, participated in the suvey, with the overall response rate of 68.3%. Altogether 915 samples were available for microscopic examination using the FEC in Western Division, whereas 995 in Central/Northern Divisions (Table 1). The age range of the participants was between 4 and 10 years, and 92.6% were either 6 or 7 years old. More male students (52.2%) were enrolled than female students (47.2%) (Table 1). The overall proportion of students being stunted, wasted, and underweight was all lower than 5% (4.7%, 4.7%, and 3.2%, respectively). The distribution of males and females, location of schools between urban and rural, source of water supply, or latrine type at schools was not significantly different between Western and Central/Northern schools, but there were a greater number of students aged 4 or 5 years, stunted, wasted, and underweight in Western schools than in Central/Northern schools with statistical significance (Table 1).

**Table 1:** Demographic characteristics and microscopic examination of gastrointestinal protozoan infection of primary school children in Fiji, 2014–2015

	Western Division	Central/Northern Divisions	*P* value	All
	(*N* = 915)	(*N* = 885)		(*N* = 1,800)
Sex			0.568	
Female	46.8%	48.9%		47.8%
Male	53.2%	51.1%		52.2%
Age			0.006	
4–5	4.2%	0.3%		2.5%
6	48.1%	46.7%		47.5%
7	43.5%	47.2%		45.1%
8–10	4.2%	5.8%		4.9%
Nutritional status*				
Stunted (< −2 SD HAZ)	7.8%	1.0%	0.000	4.7%
Wasted (< −2 SD BAZ)	6.8%	2.1%	0.033	4.7%
Underweight (< −2 SD WAZ)	4.8%	1.2%	0.022	3.2%
School location			0.516	
Urban	26.1%	16.8%		22.0%
Rural	73.9%	83.2%		78.0%
Source of water supply at school			0.487	
Fiji Water authority	46.4%	35.5%		41.5%
Others	53.6%	64.5%		58.5%
Latrine type at school			0.799	
Pour-flush	96.3%	97.3%		96.7%
Others	3.7%	2.7%		3.3%
*Giardia* infection status			0.094	
Overall prevalence (%) (95% CI)	2.2 (1.1–4.4)	0.8 (0.3–2.2)		1.6 (0.9–2.9)
Any protozoa† infection status			0.825	
Overall prevalence (%) (95% CI)	4.9 (2.6–8.8)	4.4 (2.3–8.2)		4.7 (3.0–7.2)

* BAZ = body mass index for age *z*-score; CI = confidence interval; HAZ = height-for-age *z*-score; SD = standard deviation; WAZ = weight-for-age *z*-score.

† Any protozoa also include *Entamoeba coli*, *Entamoeba histolytica*/*dispar*, *Iodamoeba butschlii*, and *Blastocystis* spp.

Based on the FEC, overall, 4.7% of examined stool samples were positive for any parasitic protozoa and 1.6% of samples were identified as having *Giardia* cysts (Table 1). Other protozoan species discovered were *Entamoeba coli*, (2.7%), *Entamoeba histolytica*/*dispar* (0.6%), *Iodamoeba butschlii* (0.1%), and *Blastocystis* spp. (0.1%), singly or in combination with other species. No significant statistical differences regarding any protozoa or *Giardia* infection prevalence between Western and Central/Northern surveys were observed (Table 1).

As per the associated factors, gender or age groups (Table 2) were not associated with increased prevalence levels of any protozoa or *Giardia* infection. Students with stunting showed slightly higher any protozoa and *Giardia* infection prevalence, but there was no statistical significance. Wasting or underweight was not associated with the elevated prevalence of any protozoa or *Giardia* infection either. When the classification of schools was considered depending on the school’s location, any protozoan infection prevalence was higher in rural schools and in schools without Fiji Water Authority supply or pour-flush latrines. By contrast, *Giardia* infection prevalence was higher in urban schools, as well as in schools with pour-flush latrines, but none of them showed statistical significance.

**Table 2:** Prevalence of *Giardia* and any protozoan infection of primary school children in Fiji according to demographic characteristics

	*Giardia* infection (%) (95% CI)	*P* value	Any protozoan infection (%) (95% CI)	*P* value
Sex		0.902		0.938
Female	1.6 (0.6–4.1)		4.6 (2.3–8.2)	
Male	1.7 (0.9–3.3)		4.7 (2.7–8.1)	
Age		0.725		0.659
4–5	0.0		0.0	
6	1.8 (0.8–3.8)		4.4 (2.4–7.8)	
7	1.8 (1.0–3.4)		5.1 (3.1–8.4)	
8–10	0.0		3.7 (0.9–13.6)	
Stunted (< −2 SD HAZ)		0.925		0.544
Yes	1.8 (0.2–11.5)		6.1 (2.2–16.3)	
No	1.6 (0.9–2.8)		4.6 (3.0–7.1)	
Wasted (< −2 SD BAZ)		0.508		0.243
Yes	0.0		2.2 (0.6–7.5)	
No	1.7 (1.0–3.0)		4.8 (3.0–7.4)	
Underweight (< −2 SD WAZ)		0.522		0.313
Yes	0.0		0.0	
No	1.7 (0.9–3.0)		4.8 (3.1–7.4)	
School location		0.694		0.517
Urban	2.0 (0.6–6.0)		3.7 (1.7–7.9)	
Rural	1.5 (0.8–3.0)		4.9 (3.0–8.1)	
Source of water supply at school		0.645		0.111
Fiji Water Authority	1.4 (0.5–3.4)		3.1 (1.7–5.5)	
Others	1.8 (0.9–3.7)		5.8 (3.4–9.8)	
Latrine type at school		0.480		0.130
Pour-flush	1.6 (0.9–2.9)		4.5 (2.8–7.1)	
Others	0.9 (0.1–5.0)		9.3 (3.9–20.3)	

BAZ = body mass index for age *z*-score; CI = confidence interval; HAZ = height-for-age *z*-score; SD = standard deviation; WAZ = weight-for-age *z*-score.

**Figure 2.**
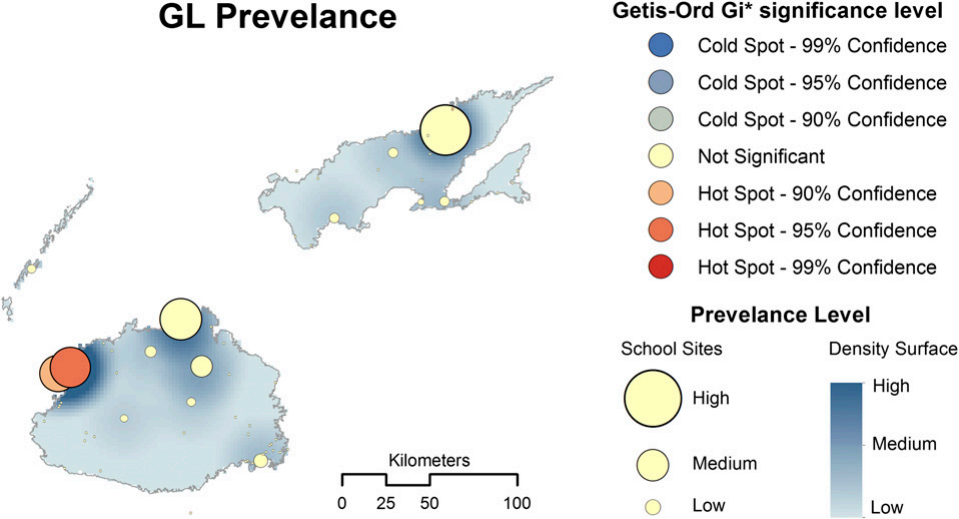
Spatial clustering trends and density distribution of *Giardia* infection among primary school children of two main islands of Fiji. This figure appears in color at www.ajtmh.org.

The school-level prevalence of *Giardia* infection ranged from 0.0% to 15.6%, and a third of 30 schools in Western Division had *Giardia-*infected cases compared with just under a fifth of Central/Northern Division schools (Figure 1). Using the Getis-Ord Gi* method (optimized hotspot analysis using inverse distance), we identified a statistically significant higher prevalence of *Giardia* infection in schools located around the Lautoka city council of the Western Division (*Z*-score = 3.36, *P* value < 0.05). Figure 2 shows the results of the Gi* analysis identifying a global trend with a surface density map (kernel density).

From the molecular analysis, the overall prevalence of *Giardia* infection in Western Division was up to 22.4% (95% CI: 16.6–32.0%), and we were able to confirm the existence of *E. histolytica* (prevalence 2.3%) and the absence of C*ryptosporidium parvum* among examined stool samples.

## DISCUSSION

Having a better appraisal of gastrointestinal protozoan infection is important but continues to be problematic owing to present diagnostic difficulties in both operational and reference diagnostic settings.^23^ Previously, on Fiji, only a few studies have been performed, but with limited focus on gastrointestinal protozoan infection, and were undertaken long ago during 1960–1980s with variable results.^15^ In 1968, a survey conducted in a rural village reported *Giardia lamblia*, with prevalence levels of 5.4%,^16^ whereas another survey near Sigatoka Valley in 1982 reported *Giardia* infection among children younger than 15 years, with prevalence levels of 1–5%.^24^

Today, in this cross-sectional approach among first- and second-grade school children in 69 schools by coproscopy, it seems that gastrointestinal protozoan infection is not rare on the two main islands of Fiji (Figure 1). Given the insensitivity of single stool sampling, the actual prevalence could be higher, and it would have been better to attempt further fecal sampling such as three consecutive day stool samples,^25^ but was not possible due to the logistical challenges in the field. Nevertheless, our findings were further confirmed by real-time PCR, showing that the *Giardia* infection prevalence of every 10th sample collected in Western reached up to 22.4%, as similarly observed in other recent studies in different parts of the world.^25^ We believe that this is mainly due to the higher sensitivity and specificity of the applied molecular technique,^21^ but it could also reflect the high level of the *Giardia* infection endemicity at the time of the survey.

We found that the spatial distribution of *Giardia* infection at the school level across two islands was not uniform but was clustered, and cases of the *Giardia* infection were grouped at certain schools, mostly around urban centers such as Lautoka, the second biggest city in Fiji, and Ra town in Western Division. From the spatial analysis, we were able to confirm that there was a real hotspot of *Giardia* infection in these areas. This is unexpected, given that *Giardia* infection is thought to result mainly from the use of unprotected water sources^26^ of which their distribution is more prevalent in rural settings.^27^ As a study in the postearthquake camps of Colombia indicated that giardiasis could emerge during events, which alter the existing water and sanitary conditions,^28^ and episodes of flooding and heavy water runoff can subsequently contaminate water and foods with *Giardia* cysts from an infected human or from animal wastes.^28,29^ Thus, it may be possible that *Giardia* infection in these major urban centers originated from the contaminated water and foods by the floods, caused by Cyclone Kofi in February 2014,^30^ which occurred just before our stool samples were collected. In this regard, we propose that enhanced surveillance efforts, including water quality testing in the disaster-affected areas as part of the preparedness plan are needed to explore whether there was possible contamination of water sources or any increased level of the endemicity of the *Giardia* infection. These should be implemented urgently whenever there are major events that can alter the water and sanitary conditions in future.

We also found that *Giardia* infection prevalence was higher among those without wasting (weight-for-height Z-scores [WHZ] < −2) or underweight (WAZ < −2). Interestingly, other studies in Ethiopia, Brazil, and Iran^31–33^ showed that children with *Giardia* infection were more likely to have lower WHZ scores and wasting compared with children without the infection. This seems to be logical, given that most of the infection would be transient than persistent, and wasting is a sign of acute undernutrition.^10^ These studies also showed that children with *Giardia* infection can have lower WAZ scores, but not likely to be classified as underweight.^31–33^ Although it was not statistically significant, our finding is rather surprising, but with a small number of events, it is challenging to make inference on the real association, which would not allow adjusting other covariates. Similar trends were observed for any protozoan infection prevalence with wasting and being underweight, but among those who were stunt (HAZ < −2), any protozoan infection prevalence was higher, which is consistent with previous studies.^34^

As for other environmental factors associated with the infection, we found that school-level water sources from the non-Fiji Water Authority and nonpour-flush latrine types are associated with higher any protozoan infection prevalence, although the association was not strong enough to have statistical significance. This is in line with previous studies, as having Fiji Water Authority supply would imply that all drinking water will be ideally pretreated with chemicals and sedimentation, filtered, and disinfected,^35^ which will lower the probability of water contamination with protozoan cysts. Also, pour-flush toilets at schools had protective effects of the infection, as it is known to be associated with environmental contamination.^27^ However, attention should be paid to interpret this finding, as recent statistical modeling showed strong evidence of protozoan contamination of shallow groundwater from pour-flush latrines within 15 m.^36^

Except *Giardia* infection prevalence in Western Division assessed by real-time PCR, gastrointestinal protozoan infection prevalence levels in our study are lower than those in recent surveys targeting school children with similar methods in urban^37^ and rural areas in Iran^14^ with better standards of living at the national level.^38^ These findings suggest that the epidemiologic profiles of gastrointestinal protozoan infection here could differ greatly by the local factors such as being urban or rural and justify epidemiologic investigation on protozoan infection using more sensitive diagnostic techniques such as real-time PCR when the settings differ.

Although we believe that our study is the first attempt to use LF TAS as an access platform to assess the protozoan infection, it is not the first case to use LF TAS for stool collection platforms.^39^ In addition, as our study was designed and conducted before the guideline for assessing the epidemiology of STH during TAS became available,^40^ there are several differences in the survey design and the stool collection framework. First, our sample size of stool collection was greater than what is currently recommended, because of the fact that we have sampled 10 urban and rural schools in each LF TAS EU and also invited all students to participate rather than subsampling them. We believed that we have benefited from this approach, given that the protozoan infection could be clustered at the school level with the overall low-level prevalence (< 5%). Also, the age groups of stool sample collections differed, as most of our participants were 6–7 years old in comparison with 8–10 years old as recommended in the guideline. We opted for this age group with the best intention to use the small number of the survey team members and to minimize any additional workload from having two different target groups for LF TAS and stool collection, but it may be interesting to learn whether the actual burden of the protozoan infection would differ between these two different target populations.

This study has a number of limitations. As described previously, we may have underestimated the true prevalence, as we had relied on the detection of protozoan cysts by microscopy only from a single specimen per person. Given that microscopic examination of protozoan infection is time consuming and dependent on the operator’s skills and expertise, newly available antigen-based detection methods using rapid detection tests could be attractive alternatives.^41^ However, we tried to overcome this in the Western Division survey by adding real-time PCR as a quality control tool. In addition, having only first- and second-grade students in the sample may not reflect the actual epidemiologic profile of these protozoan infection among school children on Fiji, considering that broader age groups may have impacts on the risk of being infected.^25^ Another similar design of the survey with a wide range of age groups may be warranted to have a more representative picture.

With the total number of school-aged children in the study areas up to 200k,^12^ several hundreds of children could have been infected with gastrointestinal protozoa including *Giardia* spp. Although not all the infected children would develop morbidity, it is likely that in schools with these infections, there have been occasions of water or food being contaminated, which is known to be a source of the disease.^27^ Thus, it may be necessary to establish the overall public health impact of the infection in the country and what would be a core set of interventions for water, sanitation and hygiene improvement at the school level, to break the transmission cycle of these protozoan infections. Considering that the *Giardia* infection prevalence levels were even higher in urban areas in our study and even several hotspots existed, greater attention should be paid to urban and rural areas.

## CONCLUSION

By adding the FEC and real-time PCR to the LF TAS survey, we were able to shed new light on the distribution of gastrointestinal protozoan infection including *Giardia* spp. across the island. Using TAS as an access platform for surveillance of protozoan infection was convenient, and the introduction of other diagnostic techniques such as DNA-based methods using archived stool samples should be more actively pursued. Spatial analysis using the Getis-Ord Gi* method highlighted that schools with a high prevalence of *Giardia* infection were clustered around in the urban areas of the Western Division, possibly because of contaminated water or foods after the floods caused by Cyclone Kofi. Enhanced surveillance efforts should be considered in the disaster-affected areas to explore whether there is possible contamination of water sources or any increased level of the endemicity of *Giardia* infection.
